# 

**Published:** 1892-04-30

**Authors:** 


					? PRICE TWOPENCE.
N.?'292, Vol.XII?April 30th, 1892.
^8irter?d Port Offlee u a Hswcpaper for
"^Unuuion in the United Kingdom and Almad.l
^r*ix? owwi,
WITH EXTRA NURSING SUPPLEMEf
Per Annum, 8s. 6d.( post free.
Monthly Parts, 6d.; post free, 8d.
Ditto per Annum, 8s., post free.
No. 292. Vol. XII.] Saturday,
April 30th, 1892.
[Twopence.

				

